# Narrow Band Imaging by Novice Raters After Short Training: A Pilot Feasibility Study of Diagnostic Accuracy and Repeatability

**DOI:** 10.1111/odi.70357

**Published:** 2026-04-26

**Authors:** Matteo Zotti, Katia Rupel, Edgar Andrea Keller, Griselda Pedroso Acosta, Roberto Di Lenarda, Matteo Biasotto, Giulia Ottaviani

**Affiliations:** ^1^ Department of Medicine, Surgery and Health Sciences University of Trieste Trieste Italy

**Keywords:** diagnostic accuracy, narrow band imaging, oral potentially malignant disorders, sensitivity and specificity, Takano classification

## Abstract

**Objective:**

To pilot the feasibility, diagnostic accuracy, and repeatability of the Takano NBI‐based IPCL classification when applied by novice raters after short formal training.

**Methods:**

Ten dental students classified 90 oral lesions shown as paired white‐light and NBI images in two sessions, 4 weeks apart. Scores were recorded on an ordinal scale (0–4), where category 0 denoted hyperplasia or clinically normal mucosa, while categories 1–4 represented increasing degrees of epithelial dysplasia or invasive disease. Histopathology served as the reference standard.

**Results:**

The 90 lesions comprised 38 hyperplastic/normal, 29 dysplastic, and 23 invasive carcinomas. Mean agreement with histology was 18.8% at T1 and 21.0% at T2, with weighted kappa values of 0.080 and 0.093, respectively. Agreement with the expert rater was 22.2% at T1 and 20.4% at T2, with weighted kappa values of 0.170 and 0.127.

**Conclusion:**

A short lecture‐based training is insufficient for reliable NBI interpretation by novice raters. These findings support the necessity for structured, competency‐based training and supervised case libraries before Takano‐based NBI assessment can be recommended in clinical practice for inexperienced practitioners.

## 1 Introduction

The early detection of oral potentially malignant disorders (OPMDs) and oral squamous cell carcinoma (OSCC) is essential to improve patient prognosis and reduce morbidity and mortality (Piazza et al. 2008; Watanabe et al. 2008). Conventional white‐light examination has limited ability to identify dysplastic or neoplastic mucosal changes, and adjunctive visualization techniques have therefore gained increasing attention (Algire et al. 1945; Berra et al. 2006; Gimbrone et al. 1972; Greenblatt and Shubi 1968; Kalas et al. 2005). Among these, narrow band imaging (NBI) enhances superficial mucosal vascular patterns by using filtered blue and green wavelengths and may improve recognition of suspicious oral lesions (Chu et al. 2012; Piazza et al. 2008; Yang et al. 2014). Systematic reviews and meta‐analyses have reported encouraging performance for NBI by expert raters, but they have also highlighted marked heterogeneity related to lesion spectrum, study design, threshold definition, image‐based versus live assessment, and operator expertise (Mendonca et al. 2022; Saraniti et al. 2021; Zhang et al. 2022).

The diagnostic rationale of NBI is based on the assessment of intrapapillary capillary loops (IPCLs). The morphological alteration of these vascular patterns, described by the Takano classification, has been associated with dysplasia and malignancy (Takano et al. 2010). The Takano classification provides an ordinal framework for grading these vascular patterns. However, most favorable reports have been generated by expert raters, whereas novice clinical implementation remains uncertain. Because diagnostic tools intended for routine practice must be interpretable beyond highly specialized operators, the reproducibility of NBI interpretation after limited training remains a clinically relevant issue.

Accordingly, the aim of this pilot feasibility study was to assess whether a short lecture‐based training is sufficient for novice raters to apply the Takano classification to oral images acquired under NBI with acceptable diagnostic agreement and repeatability. We also explored potential misclassification clustered in specific IPCL grades. The working hypothesis was that the weighted kappa values would remain low after a short training period because the purpose of this pilot study was to describe the reader performance rather than to prove clinical expert level.

## 2 Materials and Methods

### 2.1 Study Design

This was a single‐center pilot diagnostic accuracy and reader repeatability study based on retrospectively selected image pairs and prospectively trained novice raters. The study was conducted in accordance with the STARD (Standards for Reporting of Diagnostic Accuracy Studies) guidelines. The protocol was reviewed and approved by the University Ethical Committee (protocol no. 11_2024), and all enrolled participants provided written informed consent.

Novice raters—fourth‐year dental students from the Dental School of the University of Trieste (Italy)—participated in a structured training session consisting of a single 2‐h formal lecture focused on the visual assessment of NBI features according to the Takano classification of IPCLs. The training session was conducted by a faculty member with more than 10 years of experience in NBI (GO). No supervised calibration rounds, feedback sessions, or minimum competency thresholds were incorporated before image assessment.

After training, participants independently classified 90 oral lesions displayed as paired white‐light and NBI images using the original archived ordinal coding from 0 to 4. In the analytic dataset, category 0 identified hyperplasia/clinically normal mucosa, category 1 mild dysplasia, category 2 moderate dysplasia, category 3 severe dysplasia/carcinoma in situ, and category 4 invasive carcinoma. The assessment was performed twice, 4 weeks apart, to evaluate intra‐rater repeatability. Histopathological diagnosis on hematoxylin and eosin‐stained biopsy specimens served as the reference standard. Ratings were also compared with those of a single expert assessor with more than 10 years of experience in NBI technique. The analysis was conducted on this original ordinal scale; no post hoc binary dichotomization was applied.

To compile the dataset for this study, the database of the Oral Medicine and Pathology Unit (Maxillofacial and Odontostomatology Clinic, Maggiore Hospital, Trieste, Italy) was retrospectively reviewed. Two independent clinicians (GO and MZ) screened the records using predefined inclusion and exclusion criteria.

Inclusion criteria were defined as follows: (I) clinical suspicion of OPMDs at first examination; (II) availability of focused image pairs acquired under both white‐light and NBI, with clear visualization of the IPCL network; and (III) histopathological diagnosis obtained by incisional oral biopsy. Exclusion criteria were: (I) previous diagnosis of OPMDs at the same or different intraoral sites; (II) prior surgical treatment for OSCC; and (III) previous chemotherapy and/or head and neck radiotherapy for OSCC. Because this was a retrospective image‐based pilot study, ratings were performed on static images rather than during live clinical examination, and no external validation cohort was available.

Overall, the study was designed as a pilot reader exercise to estimate the feasibility of novice use of Takano‐based NBI interpretation, rather than to validate a definitive clinical threshold for routine practice.

### 2.2 Statistical Analysis

Primary outcomes were agreement with histology at T1 and T2 and intra‐rater repeatability between sessions. Agreement with the expert assessor was analyzed as a secondary benchmark. For each rater, percentage agreement with histology and with the expert assessor was calculated together with exact *n*/*N* values and 95% confidence intervals.

The percentages of agreement between each rater and the reference standard (within each session) were calculated, along with the percentages of repeatability between the two sessions (T1 and T2) and within each enrolled rater.

Because the analytic coding was ordinal, the degree of agreement was quantified with linearly weighted kappa coefficients and 95% confidence intervals. Linear weighting was chosen a priori because one‐grade disagreements were considered less severe than wider errors while avoiding the stronger penalty assigned by quadratic weighting in this small pilot sample. Grade‐specific disagreement from the reference standard and from the expert assessor was summarized as the order of grade‐apart difference across the full 0–4 scale.

Within each session, overall inter‐rater concordance was assessed by Kendall's W coefficient of concordance.

SPSS version 29.0 (IBM Corp., Armonk, NY, USA), MedCalc version 12.3.3.0 (MedCalc Software, Mariakerke, Belgium), and Comprehensive Meta‐Analysis version 2 (Biostat, Englewood, NJ, USA) were used to perform the statistical analyses. A *p* value less than 0.05 was used for rejection of the null hypothesis.

## 3 Results

A total of 10 novice raters were included in the original analytic dataset. Each participant classified the same dataset of 90 paired lesions in two sessions, yielding 1800 intended ordinal ratings overall.

The histopathological reference distribution across the 90 archived cases is reported in Table 1. The set included 38 hyperplastic/clinically normal lesions (42.2%), 29 dysplastic lesions overall (17 mild, 5 moderate, and 7 severe/carcinoma in situ; 32.2%), and 23 invasive carcinomas (25.6%). Representative NBI examples spanning the full reference spectrum are shown in Figure 1. Mean agreement with histology across novice raters was 18.8% at T1 and 21.0% at T2 (Table 2). The corresponding overall weighted kappa coefficients were 0.080 (95% CI 0.035–0.125) and 0.093 (95% CI 0.064–0.122), respectively. Agreement with the expert rater was slightly higher, with mean percentage agreement of 22.2% at T1 and 20.4% at T2 and weighted kappa values of 0.170 (95% CI 0.136–0.203) and 0.127 (95% CI 0.094–0.160), respectively (Table 3).

**TABLE 1:** Histopathological reference distribution of the 90 archived lesions included in the reader‐performance pilot.

Reference category	*n*	%
0—Hyperplasia/clinically normal mucosa	38	42.2
1—Mild dysplasia	17	18.9
2—Moderate dysplasia	5	5.6
3—Severe dysplasia/carcinoma in situ	7	7.8
4—Invasive carcinoma	23	25.6
Overall mean	90	100

**FIGURE 1:**
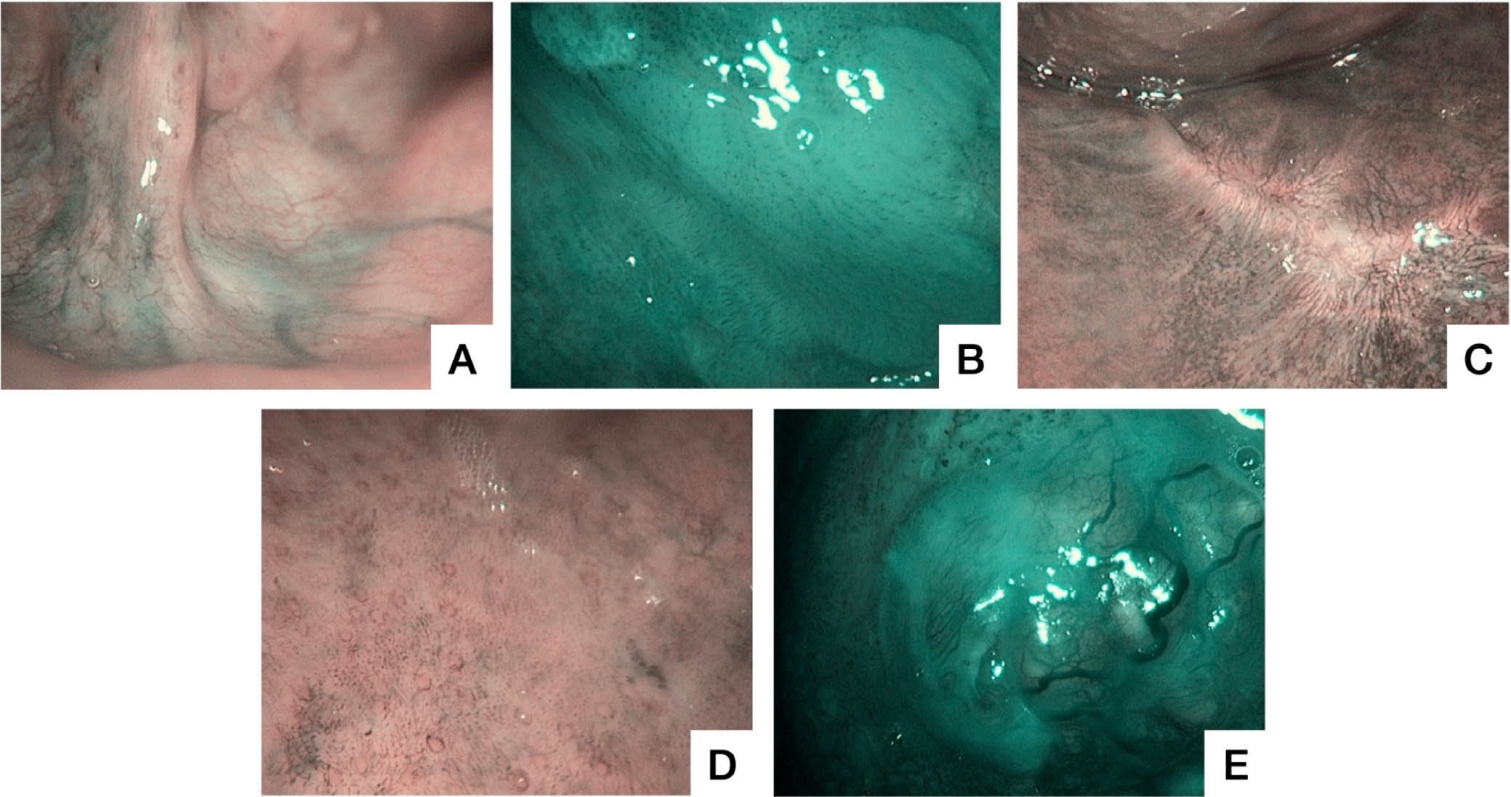
Representative NBI examples in the analytic dataset: (A) hyperplasia/clinically normal mucosa, (B) mild dysplasia, (C) moderate dysplasia, (D) severe dysplasia/carcinoma in situ, and (E) invasive carcinoma.

**TABLE 2:** Per‐rater percentage agreement with histology and the expert rater at T1 and T2, and intra‐rater agreement between sessions, reported with 95% confidence intervals.

Rater	With histology		With main rater		*n* (%)
	T1 *n*/*N* (%; 95% CI)	T2 *n*/*N* (%; 95% CI)	T1 *n*/*N* (%; 95% CI)	T2 *n*/*N* (%; 95% CI)	
1	14/90 15.6% (9.5–24.4)	14/90 15.6% (9.5–24.4)	18/90 20.0% (13.0–29.4)	11/90 12.2% (7.0–20.6)	34/90 37.8% (28.5–48.1)
2	22/90 24.4% (16.7–34.2)	24/90 26.7% (18.6–36.6)	21/90 23.3% (15.8–33.1)	27/90 30.0% (21.5–40.1)	46/90 51.1% (41.0–61.2)
3	17/90 18.9% (12.1–28.2)	19/90 21.1% (14.0–30.6)	18/90 20.0% (13.0–29.4)	14/90 15.6% (9.5–24.4)	41/90 45.6% (35.7–55.8)
4	12/90 13.3% (7.8–21.9)	18/90 20.0% (13.0–29.4)	22/90 24.4% (16.7–34.2)	17/90 18.9% (12.1–28.2)	25/90 27.8% (19.6–37.8)
5	18/90 20.0% (13.0–29.4)	19/90 21.1% (14.0–30.6)	17/90 18.9% (12.1–28.2)	20/90 22.2% (14.9–31.8)	44/90 48.9% (38.8–59.0)
6	18/90 20.0% (13.0–29.4)	19/90 21.1% (14.0–30.6)	21/90 23.3% (15.8–33.1)	15/90 16.7% (10.4–25.7)	33/90 36.7% (27.4–47.0)
7	20/90 22.2% (14.9–31.8)	22/90 24.4% (16.7–34.2)	27/90 30.0% (21.5–40.1)	28/90 31.1% (22.5–41.3)	52/90 57.8% (47.5–67.5)
8	17/90 18.9% (12.1–28.2)	16/90 17.8% (11.2–26.9)	16/90 17.8% (11.2–26.9)	15/90 16.7% (10.4–25.7)	68/90 75.6% (65.8–83.3)
9	10/90 11.1% (6.1–19.3)	20/90 22.2% (14.9–31.8)	22/90 24.4% (16.7–34.2)	25/90 27.8% (19.6–37.8)	36/90 40.0% (30.5–50.3)
10	21/90 23.3% (15.8–33.1)	18/90 20.0% (13.0–29.4)	18/90 20.0% (13.0–29.4)	12/90 13.3% (7.8–21.9)	42/90 46.7% (36.7–56.9)
Overall mean	18.8%	21.0%	22.2%	20.4%	46.8%

*Note:* Data are presented as *n*/*N* and percentage agreement with 95% confidence intervals for each rater. Overall values are means of percentages across raters. *N* = 90 lesions.

**TABLE 3:** Per‐rater weighted kappa coefficients for agreement with histology and the expert rater at T1 and T2, and for intra‐rater repeatability between sessions.

Rater	With histology		With main rater		Intra‐rater agreement T1–T2 mean (95% CI)
	T1 mean (95% CI)	T2 mean (95% CI)	T1 mean (95% CI)	T2 mean (95% CI)	
1	0.061 (0–0.180)	0.107 (0–0.226)	0.152 (0.039–0.265)	0.095 (0–0.216)	0.386 (0.257–0.515)
2	0.062 (0–0.196)	0.053 (0–0.201)	0.149 (0.049–0.248)	0.171 (0.057–0.285)	0.557 (0.438–0.676)
3	0.150 (0.030–0.269)	0.175 (0.063–0.287)	0.167 (0.064–0.271)	0.107 (0.022–0.193)	0.434 (0.299–0.568)
4	0.039 (0–0.165)	0.104 (0–0.218)	0.195 (0.080–0.311)	0.060 (0–0.169)	0.164 (0.017–0.310)
5	0.147 (0.022–0.272)	0.166 (0.044–0.287)	0.118 (0–0.236)	0.150 (0.032–0.268)	0.537 (0.426–0.648)
6	0.191 (0.064–0.318)	0.135 (0.004–0.266)	0.271 (0.157–0.385)	0.128 (0.028–0.229)	0.362 (0.232–0.493)
7	0.101 (0–0.245)	0.154 (0.015–0.293)	0.224 (0.094–0.355)	0.269 (0.153–0.384)	0.635 (0.522–0.748)
8	0.069 (0–0.191)	0.053 (0–0.169)	0.147 (0.053–0.242)	0.128 (0.042–0.214)	0.801 (0.723–0.879)
9	0.003 (0–0.121)	0.137 (0.008–0.266)	0.155 (0.046–0.263)	0.177 (0.057–0.296)	0.385 (0.241–0.528)
10	0.129 (0.006–0.251)	0.109 (0.025–0.193)	0.157 (0.072–0.243)	0.100 (0.037–0.162)	0.260 (0.126–0.394)
Overall	0.080 (0.035–0.125)	0.093 (0.064–0.122)	0.170 (0.136–0.203)	0.127 (0.094–0.160)	0.456 (0.326–0.586)

*Note:* Overall results are presented as means (95% CI) across raters. *N* = 90 lesions.

Intra‐rater repeatability between T1 and T2 reached an overall mean agreement of 46.8%, corresponding to a weighted kappa coefficient of 0.456 (95% CI 0.326–0.586). Agreement varied across raters, ranging from 27.8% to 75.8%, indicating moderate stability of individual judgments over time but no evidence of consistently high performance after brief training.

The grade‐specific disagreement analysis showed that most errors were one grade apart from the reference standard or from the expert rater (Table 4). By contrast, disagreements spanning three or four Takano grades were uncommon. The lowest disagreement values were observed for reference category 4, where both histology‐based and expert‐based comparisons yielded the smallest proportions of discordant ratings across sessions.

**TABLE 4:** Grade‐specific disagreement between novice raters and the reference standard according to the recording sessions T1 and T2, for the whole group of novice raters.

IPCL grade	Disagreement, grade apart	With histology		With main rater	
		T1 mean (min–max)	T2 mean (min–max)	T1 mean (min–max)	T2 mean (min–max)
0	1	23.9% (5.3–44.7)	20.0% (7.9–36.8)	22.5% (5.5–38.2)	20.4% (7.3–40)
	2	30.3% (15.8–44.7)	26.8% (10.5–42.1)	28.9% (20–38.2)	28.7% (14.5–38.2)
	3	20.0% (10.5–31.6)	24.5% (13.2–50)	23.6% (10.9–34.5)	27.3% (14.5–46.3)
	4	12.1% (2.6–15.8)	12.4% (2.3–26.3)	8.5% (3.6–23.6)	9.5% (0–20)
1	1	39.6% (27.8–50)	33.9% (22.2–50)	40.4% (12.5–62.5)	42.5% (12.5–75)
	2	29.7% (16.7–44.8)	33.3% (22.2–44.4)	23.0% (0–50)	22.5% (0–50)
	3	15.6% (0–38.9)	15.6% (0–27.8)	13.9% (0–25)	22.5% (0–50)
	4	—	—	—	—
2	1	52.0% (20–80)	54.0% (0–100)	53.3% (33.3–66.7)	52.5% (33.3–66.7)
	2	26.0% (0–60)	26.0% (0–80)	20.0% (8.3–33.3)	22.5% (0–41.7)
	3	—	—	—	—
	4	—	—	—	—
3	1	25.7% (0–57.1)	37.1% (14.3–57.1)	66.7% (33.3–100)	46.7% (33.3–100)
	2	22.9% (0–57.1)	17.1% (0–57.1)	3.3% (0–33.3)	6.7% (0–66.7)
	3	15.7% (0–28.6)	10.0% (0–28.6)	3.3% (0–33.3)	3.3% (0–33.3)
	4	—	—	—	—
4	1	31.4% (9.1–45.5)	28.6% (18.2–40.9)	30.8% (8.3–50)	28.3% (8.3–50)
	2	26.4% (13.6–40.9)	28.6% (9.1–45.5)	15.8% (0–33.3)	18.3% (0–41.7)
	3	11.4% (0–27.3)	9.5% (0–22.7)	6.7% (0–33.3)	5.0% (0–25)
	4	6.4% (4.5–18.2)	5.5% (0–22.7)	3.3% (0–8.3)	4.2% (0–8.3)
Overall	1	34.5 ± 11.5	34.7 ± 12.6	42.7 ± 17.6	38.1 ± 13.3
	2	27.1 ± 3.0	26.4 ± 5.9	18.2 ± 9.6	19.7 ± 8.2
	3	15.7 ± 3.5	14.9 ± 7.0	11.9 ± 9.0	14.5 ± 12.2
	4	9.3 ± 4.0	9 ± 4.9	5.9 ± 3.7	6.9 ± 3.7

*Note:* Overall results are presented as mean percentages across Takano grades. —, no cases. *N* = 90 lesions.

No binary accuracy threshold was prespecified or derived from the ordinal coding, because the purpose of the study was to assess reader agreement on the original multilevel classification rather than to estimate dichotomous sensitivity or specificity from an arbitrarily collapsed scale.

## 4 Discussion

NBI enhances visualization of superficial mucosal vascular patterns and has shown promising diagnostic performance for OPMDs and OSCC in experienced hands. The present study addressed a different question: whether brief, lecture‐only training is sufficient for novice raters to apply the Takano classification to archived oral NBI images with acceptable agreement and repeatability.

The answer of this pilot study was largely negative. Agreement with histology remained very low at both time points (weighted kappa about 0.08–0.09), and agreement with the expert rater was only slightly higher. Therefore, the key message of these data is not that NBI lacks potential value, but that its interpretation through the Takano classification is not readily transferable to novice raters trained in routine didactic settings.

This finding should be interpreted in the context of the existing literature. Previous reviews and systematic analyses have reported encouraging diagnostic performance for NBI, particularly when used by experienced clinicians and in selected clinical settings (Mendonca et al. 2022; Ota et al. 2022; Ottaviani et al. 2016; Saraniti et al. 2021; Zhang et al. 2022). At the same time, published evidence remains heterogeneous, and more recent work has emphasized that methodological factors, including lesion selection and study design, can substantially affect apparent performance. In particular, the study by Nilsson et al. should not be interpreted as supporting an exclusively experience‐based explanation for diagnostic results, because the authors also discussed relevant methodological issues related to margin assessment and case complexity (Nilsson et al. 2024).

In our study, agreement remained low after repeated exposure to the same image set, suggesting that the observed limitations were not merely due to unfamiliarity with the interface but to the greater interpretive demands of the Takano system, especially for low‐grade vascular abnormalities. In contrast, the more advanced IPCL patterns were more readily recognized, which is likely to be the most clinically meaningful training opportunity.

From a clinical and educational perspective, these pilot findings support a competency‐based training pathway rather than lecture‐only dissemination. Future curricula should incorporate annotated image libraries covering the full IPCL spectrum, explicit decision rules, supervised practice with immediate feedback, minimum performance thresholds before independent use, and periodic reassessment. This study has several limitations that must be acknowledged: the pilot sample size was small; cases were retrospectively selected from a single center; ratings were based on static images rather than live clinical examination; the rater sample was a convenience sample of dental students; training was brief and did not include calibration; and no external validation cohort was available. Taken together, these constraints mean that the present results should be interpreted as pilot evidence on feasibility and training needs rather than as definitive estimates of clinical performance.

In conclusion, Takano‐based NBI interpretation by novice raters after brief didactic exposure alone cannot yet be considered reliable for clinical use.

## Author Contributions

**Griselda Pedroso Acosta:** investigation. **Giulia Ottaviani:** conceptualization, visualization, writing – review and editing, writing – original draft. **Katia Rupel:** conceptualization, validation. **Matteo Biasotto:** conceptualization, methodology, formal analysis, project administration. **Matteo Zotti:** writing – review and editing, validation. **Roberto Di Lenarda:** supervision, data curation. **Edgar Andrea Keller:** methodology, data curation.

## Conflicts of Interest

The authors declare no conflicts of interest.

## Supporting information


**TABLE S1:** STARD checklist for reporting of studies of diagnostic accuracy (version January 2003).

## Data Availability

The data that support the findings of this study are available from the corresponding author upon reasonable request.
