# Supplementary material for: Narrow Band Imaging by Novice Raters After Short Training: A Pilot Feasibility Study of Diagnostic Accuracy and Repeatability
Source: Oral Dis. 2026 Apr 26;32(9):2725–31. doi: 10.1111/odi.70357 (PMC13752645; doi:10.1111/odi.70357)
Supplement: Supplementary file 1 [file ODI-32-2725-s001.docx]

Supplementary table

**STARD checklist for reporting of studies of diagnostic accuracy**

*(version January 2003)*

| **Section and Topic** | **Item**  **#** |  | **On page #** |
| --- | --- | --- | --- |
| TITLE/ABSTRACT/  KEYWORDS | 1 | Identify the article as a study of diagnostic accuracy (recommend MeSH heading 'sensitivity and specificity'). | **1, 2** |
| INTRODUCTION | 2 | State the research questions or study aims, such as estimating diagnostic accuracy or comparing accuracy between tests or across participant groups. | **3** |
| METHODS |  |  | **4** |
| *Participants* | 3 | The study population: The inclusion and exclusion criteria, setting and locations where data were collected. | **4** |
|  | 4 | Participant recruitment: Was recruitment based on presenting symptoms, results from previous tests, or the fact that the participants had received the index tests or the reference standard? | **4** |
|  | 5 | Participant sampling: Was the study population a consecutive series of participants defined by the selection criteria in item 3 and 4? If not, specify how participants were further selected. | **4** |
|  | 6 | Data collection: Was data collection planned before the index test and reference standard were performed (prospective study) or after (retrospective study)? | **4** |
| *Test methods* | 7 | The reference standard and its rationale. | **7** |
|  | 8 | Technical specifications of material and methods involved including how and when measurements were taken, and/or cite references for index tests and reference standard. | **5-7** |
|  | 9 | Definition of and rationale for the units, cut-offs and/or categories of the results of the index tests and the reference standard. | **6, 7** |
|  | 10 | The number, training and expertise of the persons executing and reading the index tests and the reference standard. | **5, 7** |
|  | 11 | Whether or not the readers of the index tests and reference standard were blind (masked) to the results of the other test and describe any other clinical information available to the readers. | **5** |
| *Statistical methods* | 12 | Methods for calculating or comparing measures of diagnostic accuracy, and the statistical methods used to quantify uncertainty (e.g. 95% confidence intervals). | **5-6** |
|  | 13 | Methods for calculating test reproducibility, if done. | **5-6** |
| RESULTS |  |  |  |
| *Participants* | 14 | When study was performed, including beginning and end dates of recruitment. | **4** |
|  | 15 | Clinical and demographic characteristics of the study population (at least information on age, gender, spectrum of presenting symptoms). | **4** |
|  | 16 | The number of participants satisfying the criteria for inclusion who did or did not undergo the index tests and/or the reference standard; describe why participants failed to undergo either test (a flow diagram is strongly recommended). | **4** |
| *Test results* | 17 | Time-interval between the index tests and the reference standard, and any treatment administered in between. | **4** |
|  | 18 | Distribution of severity of disease (define criteria) in those with the target condition; other diagnoses in participants without the target condition. | **4, 7, 9** |
|  | 19 | A cross tabulation of the results of the index tests (including indeterminate and missing results) by the results of the reference standard; for continuous results, the distribution of the test results by the results of the reference standard. | **Tables 1, 2** |
|  | 20 | Any adverse events from performing the index tests or the reference standard. | **Not applicable** |
| *Estimates* | 21 | Estimates of diagnostic accuracy and measures of statistical uncertainty (e.g. 95% confidence intervals). | **Tables 3, 4** |
|  | 22 | How indeterminate results, missing data and outliers of the index tests were handled. | **Not applicable** |
|  | 23 | Estimates of variability of diagnostic accuracy between subgroups of participants, readers or centers, if done. | **Tables 3, 4** |
|  | 24 | Estimates of test reproducibility, if done. | **Tables 2, 3** |
| DISCUSSION | 25 | Discuss the clinical applicability of the study findings. | **8, 9** |
